# Inflammasome activation links enteric *Salmonella* Typhimurium infection to a rapid, cytokine-dependent increase in intestinal mucin release

**DOI:** 10.1080/19490976.2024.2413372

**Published:** 2024-10-20

**Authors:** Xiao Han, Joannie M. Allaire, Shauna M. Crowley, Jocelyn J. Chan, Kelly Lau, Conghao Zhang, Simon A. Hirota, Kirk Bergstrom, Leigh A. Knodler, Bruce A. Vallance

**Affiliations:** a Division of Gastroenterology, Hepatology and Nutrition, Department of Pediatrics, BC Children’s Hospital and University of British Columbia, Vancouver, British Columbia, Canada; b Department of Physiology and Pharmacology, Snyder Institute for Chronic Diseases, University of Calgary, Calgary, Alberta, Canada; c Department of Biology, University of British Columbia, Kelowna, British Columbia, Canada; d Department of Microbiology and Molecular Genetics, Larner College of Medicine, University of Vermont, Burlington, VT, USA

**Keywords:** Salmonella, goblet cell, enteric infection, IL-22, inflammasome, mucus

## Abstract

The host restricts *Salmonella enterica* serovar Typhimurium infection of the gut via inflammasome-dependent sloughing of infected epithelial cells. Here we determined that concurrent caspase 1/11-dependent release of the goblet cell-derived mucin, Muc2, into the intestinal lumen also controls *Salmonella* burdens in infected mice. The increased release of mucins from goblet cells in the cecum and nearby proximal colon, and the subsequent thickening of the protective mucus barrier layer in the distal colon, were all dependent on the cytokines interleukin (IL)-18 and IL-22, as deficiencies in either cytokine resulted in reduced mucin secretion. Supplementation of IL-18 into IL-22 deficient mice restored mucin secretion, indicating that IL-22 acted upstream of IL-18 secretion during infection. In contrast, IL-18 and IL-22 independent signaling through Nlrp6 underlies only a modest, infection-induced increase in mucin secretion from goblet cells in the distal colon. These findings reveal that inflammasome signaling orchestrates multiple levels of protection centered on the intestinal epithelium, including pyroptosis and expulsion of infected enterocytes, as well as the release of mucins by goblet cells in the cecum and along the length of the colon. Our studies underscore the pivotal, multi-faceted role of inflammasome signaling in promoting host defense at the intestinal mucosal surface.

## Introduction

The intestinal epithelial cells (IECs) lining the gastrointestinal (GI) mucosal surface are the primary targets for an array of clinically important enteric bacterial pathogens, including *Salmonella enterica* serovar Typhimurium (*S*. Typhimurium).^1,2^ We and others have shown that invasion by this pathogen is primarily localized to the ceca of mice. *S*. Typhimurium triggers the activation of IEC-intrinsic inflammasomes in the cecum, resulting in the release of the cytokine interleukin (IL)-18,^1,2^ as well pyroptosis and expulsion of infected IECs into the intestinal lumen.^3–8^ The rapid sloughing of these cells has been shown to limit *Salmonella*’s intracellular proliferation, as well as the ability of *S*. Typhimurium to invade surrounding IECs or translocate into the underlying lamina propria. Correspondingly, inflammasome deficiency via loss of the inflammatory caspases, caspase-1 and −11, leads to heightened *S*. Typhimurium burdens in the mouse colitis model.^3–7^ However, intraepithelial *Salmonella* represent only a small fraction of the total pathogen population recovered from these mice, with the vast majority located in the intestinal lumen.^3–7^ We thus hypothesized that *S*. Typhimurium infection activates additional inflammasome-dependent responses that limit luminal pathogen burdens, while also protecting the mucosal surface.

Among the key cell types that promote intestinal mucosal defense are goblet cells, a subset of secretory IECs found throughout the GI tract, and best known for their production of the mucin family member, MUC2/Muc2. This mucin is highly O-glycosylated and stored within large granules inside goblet cells. Upon its apical secretion, Muc2 forms a thick polymeric network that provides the structural basis for the intestinal mucus barrier.^9,10^ Mucus in the colon can generally be divided into two layers: a largely impermeable barrier layer that protects underlying IECs from feces and other luminal content, and an overlying, thicker and more permeable mucus “niche” layer that is heavily colonized by commensal bacteria. Recently, the protective barrier layer was revealed to consist of two sublayers: “b1” and “b2”, based on their distinct glycosylation patterns.^11,12^ The “b1” layer, which lies above the “b2” layer, is produced by goblet cells in the proximal colon, where it encapsulates fecal pellets and migrates along with them, down the GI tract. In contrast, the “b2” mucus layer is solely secreted by goblet cells in the distal colon. The “b1” and “b2” mucus layers can be distinguished by labeling with *Maackia amurensis* lectin (MAL)-II (MALII), which binds to a sulfated glycan present in the “b2” layer only. Together these two mucus sublayers function as a formidable colonic mucus barrier that protects the underlying epithelium.^11,12^

Thus far, defining how the b1 and b2 mucus sublayers, as well as the regionally distinct goblet cells that create them, respond to enteric infections has not been explored. We do know that many parasitic, viral and bacterial infections of the GI tract are accompanied by changes in their host’s intestinal goblet cell numbers, as well as in the thickness of the intestinal mucus layers.^13,14^ For example, cecal goblet cell depletion (i.e. loss of mucin-filled goblet cells) is commonly seen during *S*. Typhimurium infection.^15^ While the mechanisms remain undefined, this may reflect the exaggerated and rapid release of mucins by goblet cells into the gut lumen, serving as a host defense mechanism.^16^ Correspondingly, Furter and colleagues used cecal explants to show that mucus architecture and organization affect the ability of *S*. Typhimurium to access the underlying epithelium.^17^ Moreover, mice lacking *Muc2* or *Muc13* show increased susceptibility to *S*. Typhimurium infections.^16^ In addition to mucins, goblet cells can release antimicrobial peptides including β-defensins, cathelicidin LL-37, ubiquitins, lysozyme as well as Reg3 lectins that possess bactericidal activities against microbes such as commensal *Escherichia coli, Staphylococcus aureus* and *Bacteroides fragilis*.^18^ It remains unclear whether goblet cells can directly respond to nearby pathogens by releasing these contents *in vivo*. However, *ex vivo* studies have identified a unique goblet cell subset, termed “sentinel goblet cells” that respond to bacterial products through activation of the Nlrp6 inflammasome and subsequent release of mucins from themselves and adjacent goblet cells, potentially to keep pathogenic bacteria away from the epithelium.^19^

In this study, we sought to define how goblet cells in the cecum, as well as the proximal and distal regions of the murine colon, respond to acute enteric infection using the *S*. Typhimurium colitis model. We also explored the role of the cytokines IL-18 and IL-22 in mediating these responses, since they are both strongly induced in this model,^20^ and both have been shown to regulate goblet cell responses in other models of GI disease.^21,22^ We observed rapid and widespread goblet cell depletion in the infected cecum, in concert with a significant increase in local luminal mucus levels, as well as a thickening of the mucus barrier layer in the distal colon. Differential staining revealed the thickened mucus barrier in the distal colon largely reflected an increase in the b1 mucus layer, originating from goblet cells in the proximal colon. These responses were found to be caspase-1/11 inflammasome-dependent, with negligible input from the Nlrp6 inflammasome. This inflammasome dependency was mediated through the cytokines, IL-18 and IL-22, as they were necessary for the infection-induced thickening of the b1 mucus layer in the distal colon. While less dramatic, the distal colon-derived b2 mucus layer also significantly increased in thickness upon infection, in part reflecting an Nlrp6 inflammasome-dependent response. Thus, during an acute enteric bacterial infection, inflammasome activation elicits a complex array of host defenses against pathogenic microbes, including mucin release, in addition to the previously described IEC pyroptosis and expulsion, and inflammatory cytokine secretion.

## Results

### S. Typhimurium infection rapidly elevates expression of the cytokines IL-18 and IL-22, along with mucin secretion, in the cecum.

**Figure 1.**
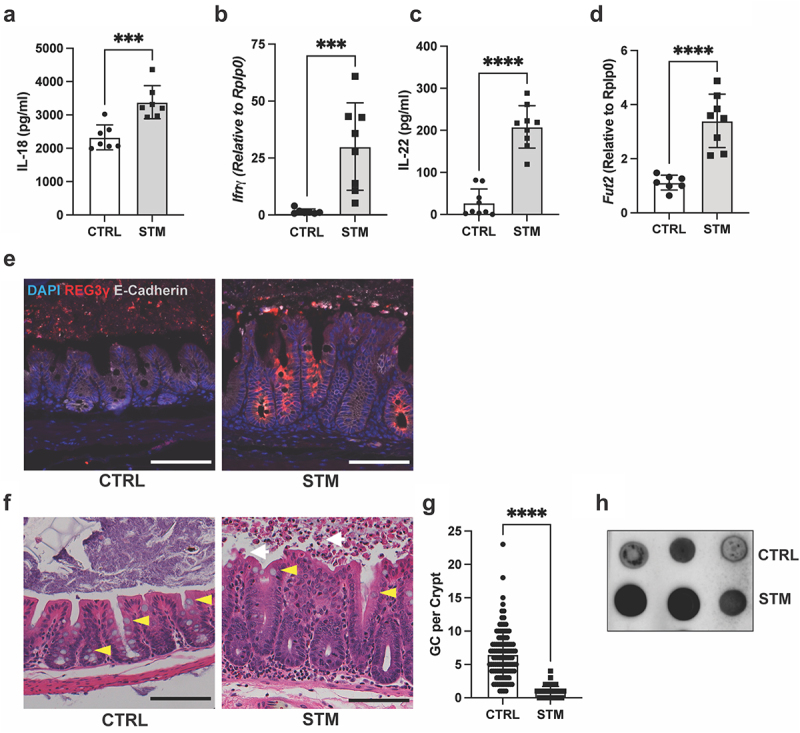
Intestinal inflammatory responses to *S*. Typhimurium infection include goblet cell depletion and mucus release in the cecum. C57BL/6 mice were orally infected with *S*. Typhimurium and cecal samples were processed at 24 h post-infection. (a) *ex vivo* release of IL-18 from the ceca of uninfected (CTRL) and infected (STM) mice was quantified by ELISA. (b) *Ifnγ* mRNA expression levels in ceca of baseline control and infected mice normalized relative to *Rplp0*. (c) *ex vivo* release of IL-22 from the ceca of uninfected (CTRL) and infected (STM) mice was quantified by ELISA. (d) *Fut2* mRNA expression. (e) Immunofluorescence staining of baseline and infected cecal tissues. Nuclei (blue), Reg3γ (red), E-cadherin (white). (f) H&E stained sections of cecum from baseline control or infected mice. Yellow arrowheads denote the presence of goblet cells. White arrows indicated sites of compromised epithelial layer and sloughed epithelial cells in lumen. (g) Quantification of goblet cell (GC) numbers in control and infected cecal crypts. Fifteen crypts were assessed per mouse. Data shown as mean ± SD. Statistical significance was determined by one-way ANOVA. ***p < 0.001; ****p < 0.0001. (h) Dot blot for Muc2. Cecal luminal contents from uninfected and infected mice were immunoblotted for Muc2. *n* = 8 for each group pooled from three independent experiments. Scale bars represent 100 μm.

The *S*. Typhimurium-induced mouse model of colitis leads to direct infection of the cecum and is characterized by the exaggerated release of an array of cytokines and antimicrobial peptides, some of which are known to promote protective IEC responses.^23^ How rapidly these responses develop is unclear, however some initial pathologies, such as inflammasome-dependent IEC sloughing are observed as early as 18–24 h post-infection (p.i.),^6^ considered the acute infection stage.^23^ To better define the cytokine milieu at this time, we orally infected streptomycin-pretreated C57BL/6 mice with *S*. Typhimurium for 24 h, noting a significant increase in release of the pro-inflammatory cytokine, IL-18, from the cecal tissues of infected mice (Figure 1a), in agreement with our earlier work.^7^ This was accompanied by increased transcription of *Ifnγ*, a cytokine that is regulated by IL-18 signaling (Figure 1b). We also observed that secretion of IL-22 from cecal tissues was significantly increased upon *S*. Typhimurium infection (Figure 1c), in concert with elevated transcription of the IL-22 regulated factor, *Fut2*, which encodes for α-1,2-fucosyltransferase, an enzyme that glycosylates proteins and lipids in the intestinal mucosa, including mucins (Figure 1d). IL-22 is also required for the direct induction of the Reg family of antimicrobial proteins.^24^ In accordance, we detected increased Reg3γ immunostaining in infected cecal tissues as compared to PBS-treated controls (Figure 1e).

Notably, both IL-18 and IL-22 can impact goblet cell function. Specifically, prior studies have shown that deleting the gene encoding IL-18 binding protein (IL18BP), a negative regulator of IL-18, leads to elevated IL-18 production and a loss of mucus-producing goblet cells in DSS-induced colitis,^22^ while IL-22 can directly induce the expression of several goblet cell mucins.^21^ Given the connection between inflammatory cytokine signaling and goblet cell function, we further investigated the impact of *S*. Typhimurium infection on this IEC subtype. *S*. Typhimurium infected C57BL/6 mice displayed severe cecal pathology, including disrupted IEC monolayer integrity, with significant numbers of IECs extruded into the lumen, along with infiltration of neutrophils and other inflammatory cells into the cecal mucosa (Figure 1f), as compared to PBS-treated controls. Goblet cell depletion was also observed by 24 h of acute *S*. Typhimurium infection (Figure 1f,g), in agreement with previous reports.^15,25^ Specifically, IECs displaying the characteristic globular morphology and mucus-filled vacuoles were rarely detected in infected ceca via H&E staining (Figure 1f), an observation that was validated by quantification of goblet cell numbers (Figure 1g).

The basis for goblet cell depletion during intestinal infection remains controversial, as it might reflect inflammation-induced damage to the goblet cells, or alternatively, the stimulated release of mucins into the intestinal lumen. *S*. Typhimurium infection leads to the luminal contents of the cecum becoming very watery, which prevented the accurate assessment and localization of Muc2 via immunostaining. Instead, we measured Muc2 protein levels in the luminal contents using a qualitative dot blot approach and detected greater amounts of Muc2 in the cecal lumen of infected mice than in PBS-treated controls (Figure 1h). This suggests that the loss of visible goblet cells in the cecum during *S*. Typhimurium infection reflects the exaggerated release of the mucin, Muc2, into the lumen. Collectively, these results indicate that the cecal mucosa rapidly responds to *S*. Typhimurium infection, with the production of antimicrobial lectins and the increased release/fucosylation of mucins as part of the host’s defensive arsenal.

### Infection leads to thickening of the b1 and b2 mucus sublayers in the distal colon

**Figure 2.**
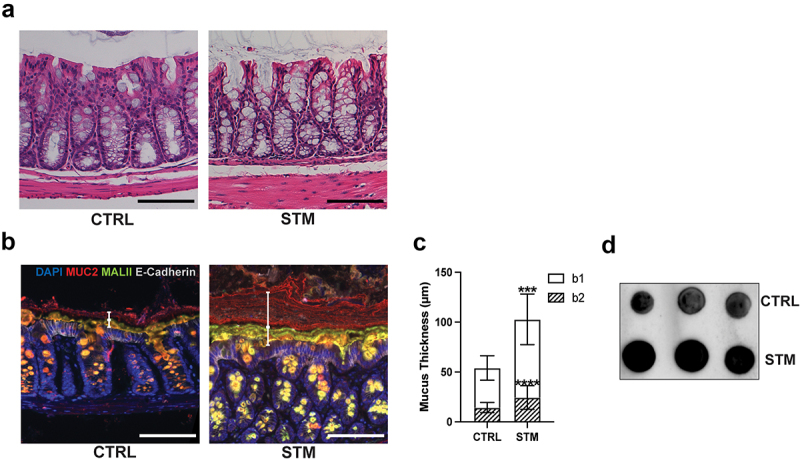
*S*. Typhimurium infection leads to increased mucus levels in the distal colon. C57BL/6 mice were orally infected with *S*. Typhimurium and distal colon samples were processed at 24 h post-infection. (a) H&E staining of distal colon from uninfected (CTRL) and infected (STM) mice. (b) Immunofluorescence staining of distal colon. Nuclei (blue), Muc2 (red), MALII (green) and E-cadherin (white). b1 and b2 mucus layers are indicated by white vertical bars. (c) Quantification of b1 and b2 mucus layer thickness. Six measurements were taken per mouse. (d) Dot blot for Muc2. Distal colon luminal content from uninfected and infected mice was immunoblotted for Muc2. Data shown as mean ± SD. Statistical significance was determined by Student’s t-test. ***p < 0.001. Scale bars represent 100 μm.

*S*. Typhimurium primarily colonizes the cecum of streptomycin-pretreated mice where it elicits severe local inflammation and pathology. Previous studies have shown the infection causes only modest tissue damage in the nearby proximal colon,^17,25^ but we found that much like in the cecum, the luminal contents of the proximal colon were too watery to measure a distinct mucus barrier. Moving aborally, while few signs of pathology were observed in formalin-fixed H&E sections of the distal colon of infected mice (Figure 2a), fecal consistency allowed us to properly image the mucus barrier. Immunostaining for Muc2 in Methacarn-fixed distal colon tissue sections (Figure 2b) revealed that *S*. Typhimurium infection led to a doubling in thickness of the mucus barrier layer as compared to PBS-treatment (Figure 2b,c). A concomitant infection-induced increase in Muc2 levels in the distal colon lumen was also observed by dot blot (Figure 2d). We used MALII staining to distinguish the site of origin of the mucus accumulation in the distal colon. Notably, acute *S*. Typhimurium infection led to a significant increase in the thickness of the b1 (MALII^−^) mucus layer (Figure 2b,c). This layer has been previously shown to arise solely from goblet cells in the proximal colon,^12^ indicating that in response to infection, these cells increase their release of Muc2 which subsequently migrates down to the distal colon. Interestingly, we also observed a lesser, but still significant, increase in the b2 (MALII^+^) layer, which is produced exclusively by goblet cells in the distal colon (Figure 2b,c), indicating that *S*. Typhimurium infection also increases mucin release by goblet cells in this region.

### Casp1/11^−/−^ *deficient mice display impaired mucin secretion during infection*

**Figure 3.**
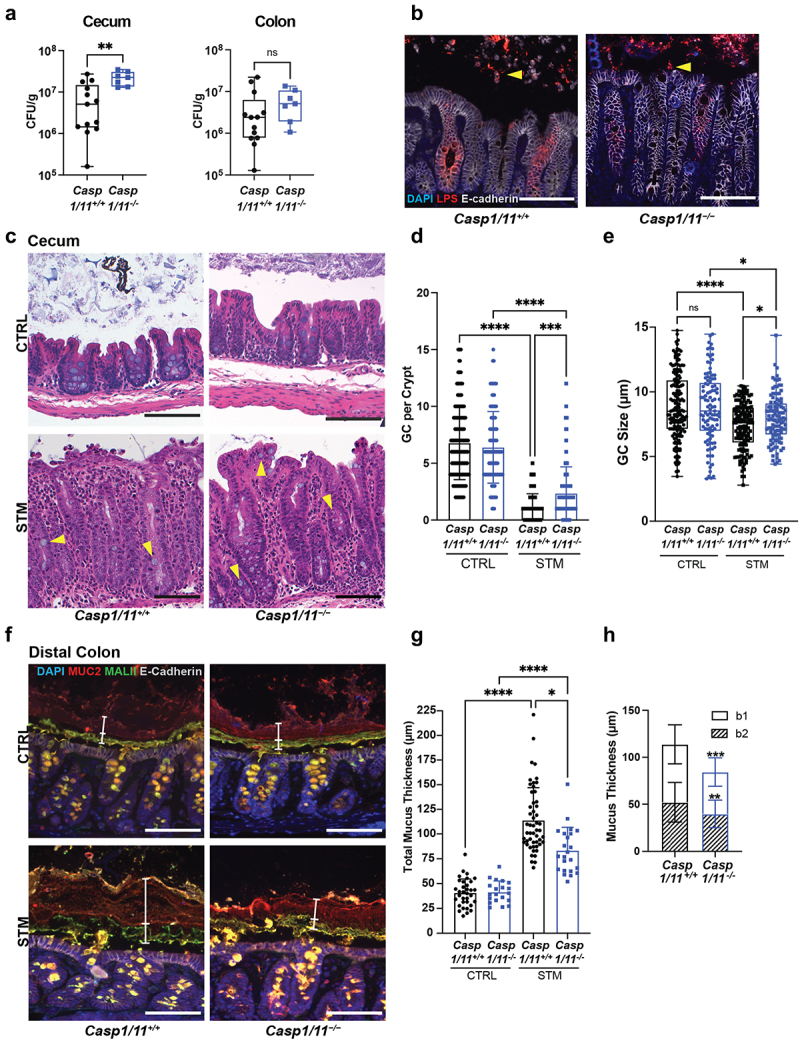
Inflammasome-deficient mice display reduced goblet cell responses and mucus secretion during *S*. Typhimurium infection. (a) Box and whisker plots of colony forming units (CFU) recovered from the ceca and colons of *Casp1/11*^*-/-*^ (blue) and *Casp1/11*^*+/+*^ (black) littermates infected with *S*. Typhimurium for 24 h. (b) Immunofluorescence staining of ceca from infected *Casp1/11*^*-/-*^ and *Casp1/11*^*+/+*^ littermates. Nuclei (blue), *Salmonella* LPS (red), E-cadherin (white). *S*. Typhimurium in lumen are indicated by yellow arrowheads. (c) H&E staining of uninfected (CTRL) and *S*. Typhimurium-infected (STM) ceca. Goblet cells are indicated by yellow arrowheads. Quantification of goblet cell (d) number and (e) diameter in control and infected cecal crypts. Fifteen crypts were quantified per mouse. (f) Immunofluorescence staining of distal colons. Nuclei (blue), Muc2 (red), MALII (green) and E-cadherin (white). b1 and b2 mucus layers are indicated by white vertical bars. (g) Quantification of total mucus thickness. (h) Quantification of b1 and b2 mucus layer thickness in infected mice. Six measurements were taken per mouse. Data shown as mean ± SD. Statistical significance was determined by one-way ANOVA. *p < 0.05; **p < 0.01; ***p < 0.001; ****p < 0.0001. Scale bars represent 100 μm.

*S*. Typhimurium infection of the mouse cecum activates inflammasome signaling involving both caspase-1 and caspase-11.^3,6,7^ These inflammatory caspases were previously implicated in the release of mucins from goblet cells in mouse colonic explants exposed to bacterial products,^19^ but a similar role has yet to be demonstrated *in vivo*. To address this, we examined cecal tissue sections processed for H&E staining from uninfected and infected *Casp1/11*^−/−^ mice and their littermate controls (*Casp1/11*^+/+^, referred to as wild-type (WT) mice). In agreement with previous studies,^3,6^ *Casp1/11*^*−/−*^ mice carried significantly higher pathogen burdens in cecal tissues than their WT littermates at 24 h (Figure 3a). Immunofluorescence staining of cecal tissues with anti-*Salmonella* lipopolysaccharide (LPS) and anti-E-cadherin antibodies showed many luminal *Salmonella* near the mucosal surface in *Casp1/11*^*−/−*^ mice but not in WT mice (Figure 3b), suggesting a role for inflammasome-associated caspases in controlling pathogen access to the epithelial layer. The infected ceca of WT mice displayed widespread goblet cell depletion (Figure 3c), with significantly fewer goblet cells visible in cecal crypts by 24 h (Figure 3d). While infection also reduced goblet cell numbers in the ceca of *Casp1/11*^−/−^ mice, it was to a lesser extent than for infected WT mice (Figure 3d). The remaining goblet cells in the ceca of infected *Casp1/11*^−/−^ mice were also significantly larger (mean diameter of 7.96 µm) than those in infected WT mice (mean diameter of 7.37 µm) (Figure 3e). Notably, there was no difference in goblet cell numbers (Figure 3d) or size (Figure 3e; mean of 8.86 µm for WT and 8.75 µm for *Casp1/11*^−/−^; *p* = 0.69) in the ceca of WT and *Casp1/11*^−/−^ mice at baseline.

In the distal colon, there were higher pathogen burdens in *Casp1/11*^−/−^ mice, albeit without statistical significance (*p* = 0.18; Figure 3a). Muc2 immunostaining of uninfected tissues showed no difference in baseline mucus layer thickness between WT and *Casp1/11*^−/−^ mice (Figure 3f,g). During *S*. Typhimurium infection, the mucus layers in the distal colons of both WT and *Casp1/11*^−/−^ mice increased (Figure 3f,g), but the mucus thickening was significantly greater in the WT mice (Figure 3f,g). We used MALII and Muc2 immunostaining to characterize the b1 and b2 mucus layers in the distal colon and define the source of the increased mucus. The thickening of the b1 and b2 layers upon *S*. Typhimurium infection was significantly less in the *Casp1/11*^−/−^ mice as compared to WT mice (Figure 3h) suggesting that inflammasome signaling impacts mucin release both close to the site of infection (proximal colon), as well as in the distal colon. Collectively, these findings indicate that *Casp1/11*^−/−^ mice are impaired in their ability to release Muc2 in response to an enteric bacterial infection.

### Infection induced Nlrp6 signaling increases mucin secretion in the distal colon

**Figure 4.**
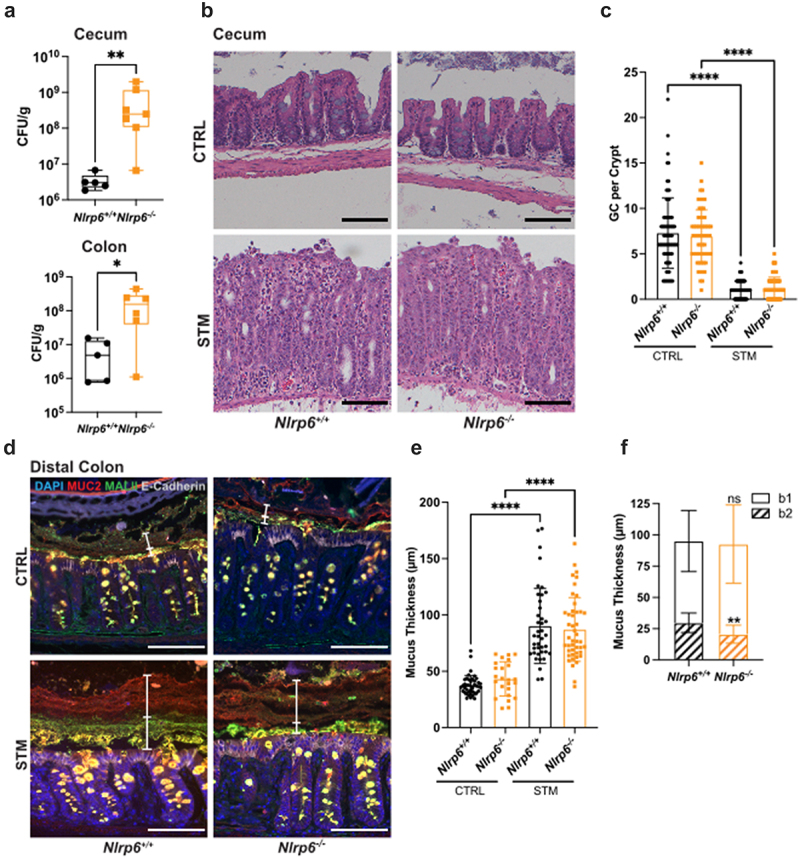
The Nlrp6 inflammasome impacts mucus secretion in the distal colon. (a) Box and whisker plots of CFU recovered from the ceca and colons of *Nlrp6*^*-/-*^ (orange) and *Nlrp6*^*+/+*^ (black) littermates infected with *S*. Typhimurium for 24 h. (b) H&E staining of cecum. (c) Enumeration of goblet cells in cecal crypts of control (CTRL) and infected (STM) mice. Fifteen crypts were quantified per mouse. (d) Immunofluorescence staining of distal colon. Nuclei (blue), Muc2 (red), MALII (green) and E-cadherin (white). b1 and b2 mucus layers are indicated by white vertical bars. (e) Quantification of total mucus thickness. (f) Quantification of b1 and b2 mucus layer thickness in infected tissues. Six measurements were taken per mouse. Data shown as mean ± SD. Statistical significance was determined by one-way ANOVA. *p < 0.05; **p < 0.01; ****p < 0.0001. Scale bars represent 100 μm.

*Nlrp6* is highly expressed by colonic epithelial cells^26^ and Nlrp6 inflammasome signaling in IECs, which involves both caspase-1 and caspase-11, has been shown to regulate Muc2 exocytosis by goblet cells in murine colonic explants.^19,27^ Notably, the Nlrp6 inflammasome is not required for baseline distal colonic mucus layer formation or function.^28^ Based on the published literature, we initially suspected that the impaired mucin secretion seen in *S*. Typhimurium-infected *Casp1/11*^−/−^ mice reflected the loss of Nlrp6 inflammasome signaling. To test this hypothesis, we infected *Nlrp6*^*−/−*^ mice and their *Nlrp6*^*+/+*^ littermate controls (WT) with *S*. Typhimurium for 24 h and assessed whether their mucosal responses differed. At 24 h p.i., much like the *Casp1/11*^−/−^ mice (Figure 3a), *Nlrp6*^*−/−*^ mice carried significantly heavier pathogen burdens in their ceca and distal colons as compared to their WT littermates (Figure 4a). Unlike the *Casp1/11*^−/−^ mice however, *Nlrp6*^*−/−*^ mice phenocopied their WT littermates in terms of infection-induced cecal pathology, with both groups displaying severely disrupted epithelial integrity, IEC sloughing and immune cell infiltration (Figure 4b). There was also no significant difference in the number of goblet cells in the cecal crypts of WT and *Nlrp6*^*−/−*^ mice either prior to, or during, *S*. Typhimurium infection (Figure 4b,c). Moreover, in the distal colon, Muc2 immunostaining revealed a mucus barrier layer of similar thickness in WT and *Nlrp6*^*−/−*^ mice, both at baseline and during *S*. Typhimurium infection (Figure 4d,e). However, assessment of the two mucus sublayers by MALII immunostaining revealed that the b2 layer in infected *Nlrp6*^*−/−*^ mice was modestly, but significantly, thinner than that seen in infected WT mice (Figure 4f). In contrast, the b1 layers from infected *Nlrp6*^*−/−*^ and *Nlrp6*^+/+^ mice were equivalent in thickness (Figure 4d–f). These data indicate that Nlrp6 signaling only mildly impacts mucin release in the distal colon during *S*. Typhimurium infection and plays no overt role in the cecum/proximal colon. Therefore Nlrp6-independent inflammasome signaling mechanisms must underlie the robust goblet cell responses at these sites during infection.

### Casp1/11^−/−^ *mice are defective for IL-18 and IL-22 secretion upon enteric infection*

**Figure 5.**
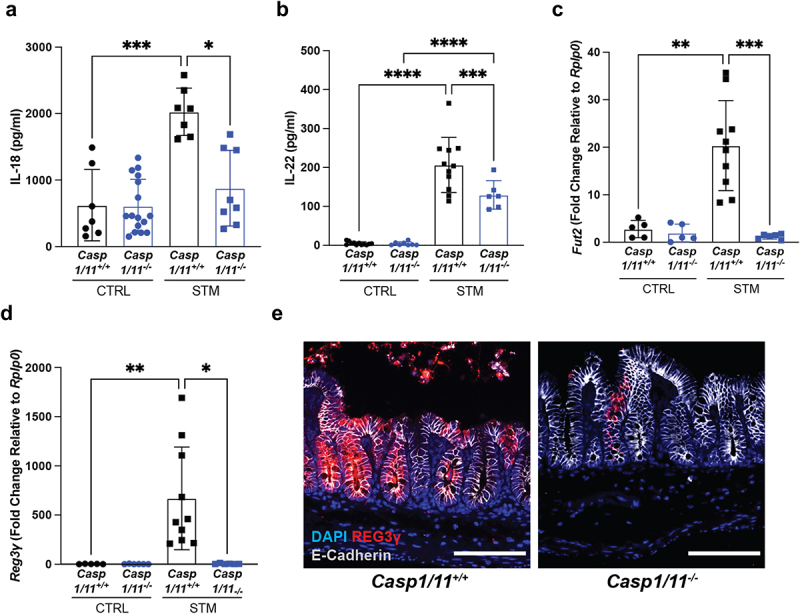
Reduced IL-18 and IL-22 secretion and downstream antimicrobial responses in inflammasome-deficient mice during *S*. Typhimurium infection. *Casp1/11*^*+/+*^ and *Casp1/11*^−/−^ were infected with *S*. Typhimurium for 24 h. *ex vivo* secretion of IL-18 (a) and IL-22 (b) from ceca of baseline control (CTRL) or infected (STM) mice was quantified by ELISA. (c) *Fut2* and (d) *Reg3γ* expression relative to *Rplp0* in ceca of infected and baseline control mice. (e) Immunofluorescence staining of ceca from infected *Casp1/11*^*+/+*^ and *Casp1/11*^−/−^ littermates. Nuclei (blue), Reg3γ (red), E-Cadherin (white). Data shown as mean ± SD. Statistical significance was determined by one-way ANOVA. *p < 0.05; **p < 0.01; ***p < 0.001; ****p < 0.0001. Scale bars represent 100 μm.

The widespread inflammasome-dependent (but largely Nlrp6-independent) mucin release from goblet cells not only in the cecum, but also in the proximal and distal colon, suggested a systemic response, likely involving soluble mediators. We investigated whether IL-18 and/or IL-22 might be involved. As noted earlier, the inflammasome-dependent cytokine IL-18 is rapidly increased during *S*. Typhimurium infection and controls mucosal inflammation kinetics.^29^ IL-18 has also been linked to goblet cell maturation and depletion in DSS-induced colitis, as well as mucin and antimicrobial peptide (AMP) secretion.^22,30^ In *ex vivo* secretion assays, we confirmed that *Casp1/11*^−/−^ mice are compromised in their ability to produce IL-18 in the cecum upon *S*. Typhimurium infection (Figure 5a).^3^ Importantly, IL-18 secretion was comparable for WT and *Casp1/11*^−/−^ mice under PBS-treated control (baseline) conditions (Figure 5a).

IL-22 is a tissue protective cytokine which exclusively targets epithelial lineages, with *Il22* being amongst the most highly upregulated genes in the ceca of *S*. Typhimurium-infected mice.^31^ Moreover, IL-22 induces the expression of a cadre of antimicrobial genes during *S*. Typhimurium infection.^32^ While inflammatory caspases are not directly required for IL-22 secretion,^33^ IL-22-dependent signaling has been closely linked to inflammasome activation and implicated in driving inflammatory changes in the intestinal epithelium, including crypt hyperplasia, fucosylation, mucin secretion as well as AMP production.^21,34,35^ We therefore quantified IL-22 in *ex vivo* secretions collected from the ceca of WT and *Casp1/11*^−/−^ mice. At baseline, both WT and *Casp1/11*^−/−^ mouse cecal tissues produced minimal levels of IL-22, but upon *S*. Typhimurium infection, IL-22 secretion dramatically increased in WT mice (Figure 5b). *Casp1/11*^−/−^ mice also showed an infection-induced increase in IL-22, but at levels significantly lower than for WT infected mice (Figure 5b). To assess the potential downstream effects of the increased IL-22 secretion, we measured *Fut2* and *Reg3γ* expression, since these genes are known to be induced by IL-22. While *Fut2* transcription was significantly increased (~10-fold) in the ceca of infected WT mice compared to baseline, it was not induced upon infection of *Casp1/11*^−/−^ mice (Figure 5c). A comparable induction pattern was observed for *Reg3γ* (Figure 5d). Correspondingly, while cecal tissues from infected WT mice showed robust and widespread Reg3γ staining, infected *Casp1/11*^−/−^ mice displayed only a few cecal crypts containing small patches of Reg3γ^+^ IECs (Figure 5e). Taken together, these results indicate that caspase-1 and −11 are involved in the infection-induced upregulation of IL-22 in the gut, consequently inducing the expression of antimicrobial C-type lectins as well as *Fut2*, which encodes for an enzyme important for fucosylation in the gut. These results also suggest that impaired IL-18 and IL-22 release could be key contributors to the defective mucus and antimicrobial defense phenotypes observed in *Casp1/11*^−/−^ mice.

### IL-18 and IL-22 stimulate mucin secretion upon acute enteric infection

**Figure 6.**
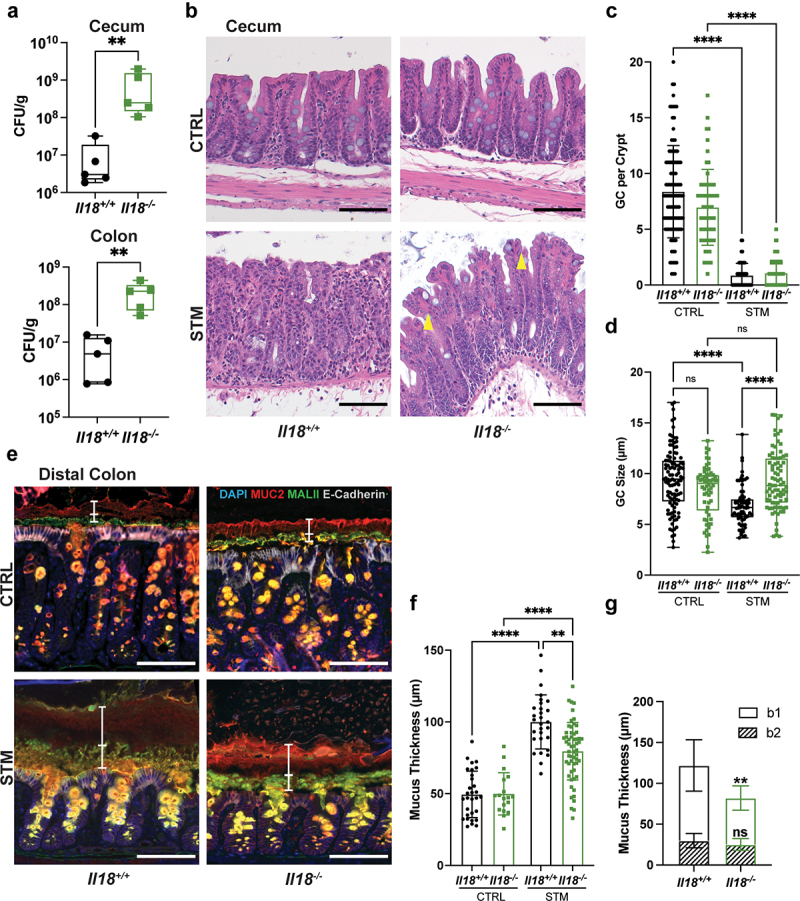
IL-18 is required to promote mucus secretion from the cecum and proximal colon in response to *S*. Typhimurium. (a) Box and whisker plots of CFU recovered from the ceca and colons of *Il18*^−/−^ (green) and *Il18*^+/+^ (black) littermates infected with *S*. Typhimurium at 24 h post-infection. (b) H&E staining of ceca. Quantification of goblet cell number (c) and diameter (d) in cecal crypts of baseline control (CTRL) and infected (STM) mice. Fifteen crypts were quantified per mouse. (e) Immunofluorescence staining of distal colon. Nuclei (blue), Muc2 (red), MALII (green) and E-cadherin (white). b1 and b2 mucus layers indicated by white vertical bars. (f) Quantification of total mucus thickness. (g) Quantification of b1 and b2 mucus layer thickness in infected tissues. Six measurements were taken per mouse. Data shown as mean ± SD. Statistical significance was determined by one-way ANOVA. **p < 0.01; ****p < 0.0001. Scale bars represent 100 μm.

As *Casp1/11*^−/−^ mice displayed a clear defect in their ability to upregulate IL-18 and IL-22 secretion at 24 h p.i., we used IL-18- and IL-22-deficient mice to test the contribution of each cytokine to the increased mucin secretion phenotype. We first infected *Il18*^*−/−*^ mice and their WT littermates with *S*. Typhimurium. Compared to WT mice, *Il18*^*−/−*^ mice carried significantly higher pathogen burdens in their ceca and distal colons at 24 h (Figure 6a). Infection led to a similar loss of cecal goblet cells (i.e. depletion) in *Il18*^*−/−*^ and WT mice (Figure 6b,c), however the goblet cells remaining in the *Il18*^*−/−*^ mice were significantly larger (mean diameter of 9.32 µm) than those in their WT littermates (mean diameter of 6.84 µm; Figure 6d). While WT and *Il18*^*−/−*^ mice showed similar mucus barrier layer thicknesses in the distal colon at baseline, *S*. Typhimurium infection promoted a significantly thicker mucus layer in the WT mice as compared to *Il18*^*−/−*^ mice (Figure 6e,f). Further characterization revealed that the disparity in mucus thickness between WT and *Il18*^*−/−*^ mice was largely driven by changes in the b1 (MALII^*−*^) layer (Figure 6g), representing mucin released from goblet cells in the proximal colon. In contrast, the thickness of the locally secreted b2 layer was similar in the two mouse strains (Figure 6g). Thus, infected *Il18*^*−/−*^ mice phenocopy the defects in cecal goblet cell depletion and impairments in the thickening of the b1 mucus layer seen in infected *Casp1/11*^−/−^ mice. We therefore conclude that caspase-1/11 inflammasome-dependent processing and release of IL-18 promotes mucin secretion in the cecum/proximal colon during acute *S*. Typhimurium infection. In contrast, IL-18 does not significantly influence mucin secretion by goblet cells in the distal colon.

**Figure 7.**
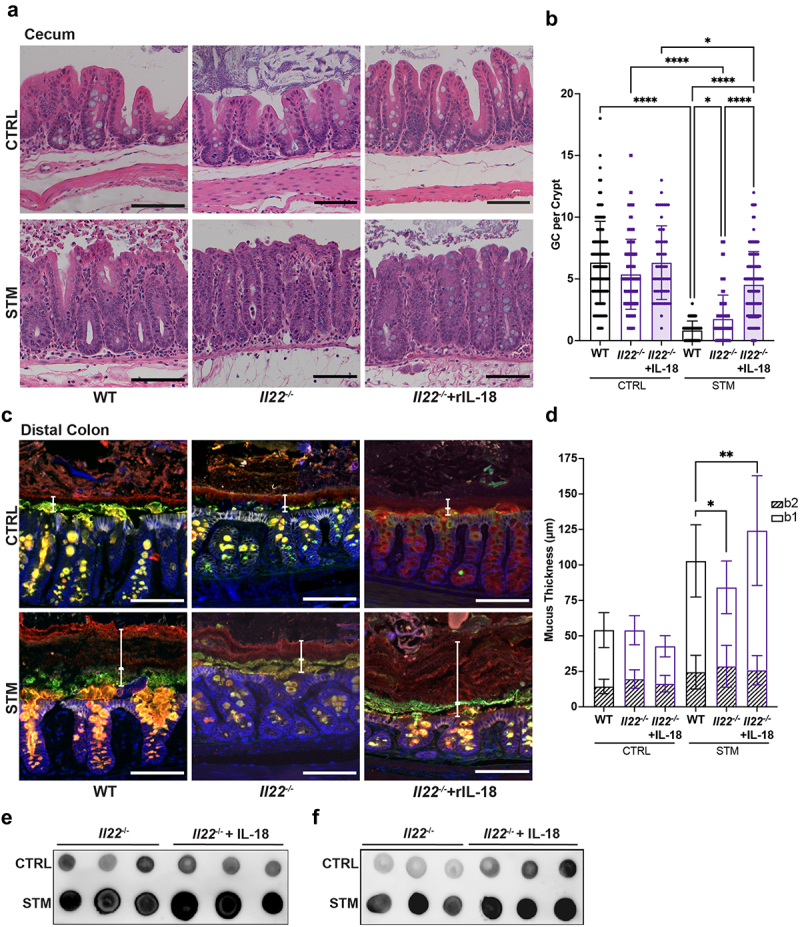
IL-18 supplementation rescues defective mucus secretion upon *S*. Typhimurium infection of IL-22 deficient mice. (a) H&E staining of ceca at 24 h post-infection. (b) Enumeration of goblet cells in cecal crypts of control (CTRL) and infected (STM) mice treated with IL-18 or PBS control. Fifteen crypts were quantified per mouse. (c) Immunofluorescence staining of distal colon. Nuclei (blue), Muc2 (red), MALII (green) and E-cadherin (white). b1 and b2 mucus layers are indicated by white vertical bars. (d) Quantification of b1 and b2 mucus layer thickness. Six measurements were taken per mouse. Dot blot for Muc2 in luminal content from (e) ceca and (f) distal colons. Data shown as mean ± SD. Statistical significance was determined by one-way ANOVA. *p < 0.05; **p < 0.01; ****p < 0.0001. Scale bars represent 100 μm.

We next tested the impact of IL-22 on mucin release during infection. Previous research has demonstrated that IL-22 promotes changes in the gut microbiota and availability of luminal nutrients that are normally subverted by *S*. Typhimurium to promote its pathogenesis.^32^ In this earlier study, *S*. Typhimurium burdens in the ceca of *Il22*^−/−^ mice were decreased compared to WT mice at 72 and 96 h, but not earlier, at 48 h p.i.^32^ Correspondingly, we found that WT and *Il22*^−/−^ mice carried similar cecal burdens of *S*. Typhimurium after 24 h of infection (∼ 5 × 10^6^ colony forming units (CFU)/gram tissue). Despite the similar pathogen burdens, *Il22*^−/−^ mice displayed significantly less cecal goblet cell depletion than WT mice upon infection, suggesting less mucin secretion into the lumen (Figure 7a,b). Immunofluorescence staining of the distal colons of *Il22*^−/−^ and WT mice for Muc2 showed that their mucus barrier layers were similar in thickness at baseline (Figure 7c,d). As expected, infection led to a modest, but significant thickening of the distal colonic mucus barrier in *Il22*^−/−^ mice, whereas the mucus barrier in infected WT mice was significantly thicker than both uninfected WT mice and infected *Il22*^−/−^ mice (Figure 7c,d). When we used MALII immunostaining to clarify the source of the mucus, it revealed that the differences between WT and *Il22*^−/−^ mice reflected a thinner b1 mucus layer in the infected *Il22*^−/−^ mice (Figure 7c,d). In contrast, the b2 mucus layer thickness was comparable for WT and *Il22*^−/−^ mice after infection (Figure 7c,d). Taken together, these findings resemble those we describe for infected *Casp1/11*^*−/−*^ mice (Figure 3) and *Il18*^−/−^ mice (Figure 6), and suggest that IL-22, upregulated in part by inflammasome activation, is also critical for promoting mucin secretion in the proximal colon, but not in the distal colon during *S*. Typhimurium infection.

### IL-22 promotes IL-18 production during S. typhimurium infection

Considerable crosstalk has been reported for IL-18 and IL-22, with the two cytokines maintaining and augmenting each other’s production during intestinal homeostasis and infection.^33,36,37^ To clarify the potential interdependence of IL-18 and IL-22 signaling during *S*. Typhimurium infection, we used *Il18*- and *Il22*-deficient mice. ELISA measurements of *ex vivo* secretions verified that the ceca of *Il18*^*−/−*^ mice failed to produce IL-18 under control or infection conditions (Fig S1A) and showed that IL-18 deficiency did not affect IL-22 release, as WT and *Il18*^*−/−*^ mice displayed comparable IL-22 secretion levels under basal and infection conditions (Fig S1B). Likewise, immunostaining of Reg3γ showed a similarly strong intensity in the cecal crypts of infected WT and *Il18*^*−/−*^ mice (Fig S1C). Thus, IL-18 is not required for the upregulation of IL-22 seen during acute enteric *S*. Typhimurium infection (24 h p.i.).

**Figure 8.**
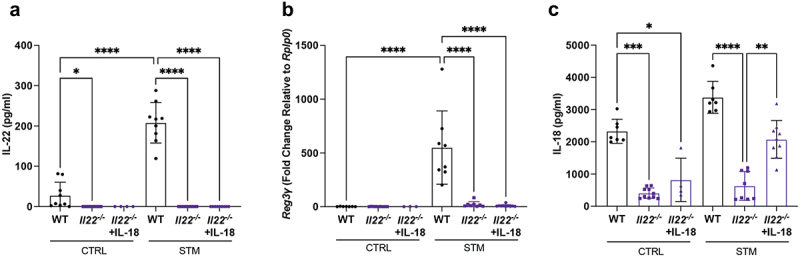
IL-18 supplementation in IL-22 deficient mice restores IL-18, but not IL-22, release. WT C57BL/6 and *Il22*^*-/-*^ mice were infected with *S*. Typhimurium for 24 h. Mice were treated with 2 µg of recombinant IL-18 (+IL-18) or PBS at 6 h post-infection via intraperitoneal injection. (a) *ex vivo* secretion of IL-22 from ceca of baseline control (CTRL) or infected (STM) mice was quantified by ELISA. (b) *Reg3γ* mRNA expression level in ceca of baseline control (CTRL) and infected (STM) mice treated with PBS or recombinant IL-18, normalized relative to *Rplp0*. (c) *ex vivo* secretion of IL-18 from ceca of baseline control (CTRL) or infected (STM) mice was quantified by ELISA. *n* ≥ 4 for each group pooled from two independent experiments. Data shown as mean ± SD. Statistical significance was determined by one-way ANOVA. *p < 0.05; **p < 0.01; ***p < 0.001; ****p < 0.0001.

For the reciprocal experiments, we used *Il22*-deficient mice. As expected, IL-22 was not secreted from the ceca of *Il22*^−/−^ mice under control (baseline) or *S*. Typhimurium infection conditions (Figure 8a). *Reg3γ* was also barely detectable in the ceca of infected *Il22*^−/−^ mice (Figure 8b). Notably, quantification of IL-18 release from the ceca of WT and *Il22*^−/−^ mice revealed severely reduced levels in *Il22*^−/−^ mice under both PBS-treated and *S*. Typhimurium-infection conditions (Figure 8c). In addition, treating C57BL/6 mice with a dosage of αIL-22 prior to bacterial infection not only reduced the mucus thickening seen in infected mice given the IgG control (Fig S2A) but also abrogated the infection-induced release of IL-22 from the cecum (Fig S2B), and decreased IL-18 production in comparison to control IgG-treated mice (Fig S2C). These findings highlight the interconnected relationship between IL-22 and IL-18 during acute enteric infection and that IL-22 acts upstream of IL-18.

We also assessed whether Nlrp6 impacted infection-induced IL-18 and IL-22 release. Both *Nlrp6*^−/−^ mice and their littermate controls displayed a dramatic, and comparable, increase in cecal IL-22 (Fig S3A) and IL-18 (Fig S3B) release during *S*. Typhimurium infection. Fucosylation, detected with the α(1,2)-fucose – recognizing lectin *Ulex europaeus* agglutinin-1 (UEA-1), was also strongly induced in both *Nlrp6*^−/−^ mice and their WT littermates (Fig S3C). These findings indicate that the modestly reduced thickening of the b2 mucus barrier layer in *Nlrp6*^−/−^ mice (Figure 4f) is not IL-18 or IL-22 dependent, and reflects mechanisms separate from those involved in the infection-induced thickening of the b1 mucus layer.

### IL-18 supplementation rescues reduced mucin secretion in IL-22 deficient mice

Based on the comparable mucin secretion phenotype seen in *Il18*^−/−^ and *Il22*^−/−^ mice during acute *S*. Typhimurium infection, and since IL-22 appears to act upstream of IL-18 following inflammasome activation by *S*. Typhimurium, we hypothesized that elevated IL-18 production directly controls mucin secretion, particularly in the cecum and proximal colon. To test this hypothesis, we infected *Il22*^−/−^ mice with *S*. Typhimurium, and then injected them intraperitoneally with PBS or 2 μg of recombinant IL-18 at 6 h p.i. While exogenous IL-18 did not affect pathogen burdens in *Il22*^−/−^ mice at 24 h (results not shown), it did significantly reduce infection-induced cecal goblet cell depletion compared to both WT and *Il22*^−/−^ mice that did not receive IL-18 treatment (Figure 7a,b). Similarly, while IL-18 supplementation did not affect the mucus barrier layer in the distal colon under baseline conditions, it did induce a significant increase in the mucus layer thickness of the *Il22*^−/−^ mice during infection (Figure 7c,d). The increased mucus largely reflected a thickening of the b1 (MALII^−^) layer, whereas the b2 mucus layer thickness was equivalent for *Il22*^−/−^ mice and IL-18 treated *Il22*^−/−^ mice (Figure 7c,d). To further assess the changes in mucus levels, we used a dot blot approach to assess Muc2 abundance in the luminal content from the cecal and distal colonic regions. Luminal content from the ceca of IL-18 treated *Il22*^−/−^ mice contained greater amounts of Muc2 protein after infection (Figure 7e), suggesting local goblet cells released more Muc2 in response to IL-18 treatment. In the distal colon, the luminal content also showed higher amounts of Muc2 (Figure 7f), largely reflective of mucin secretion from the proximal colon that had traveled aborally. Taken together, these results indicate that IL-18 promotes mucin secretion from goblet cells in the cecum and proximal colon, even in the absence of IL-22.

## Discussion

**Figure 9.**
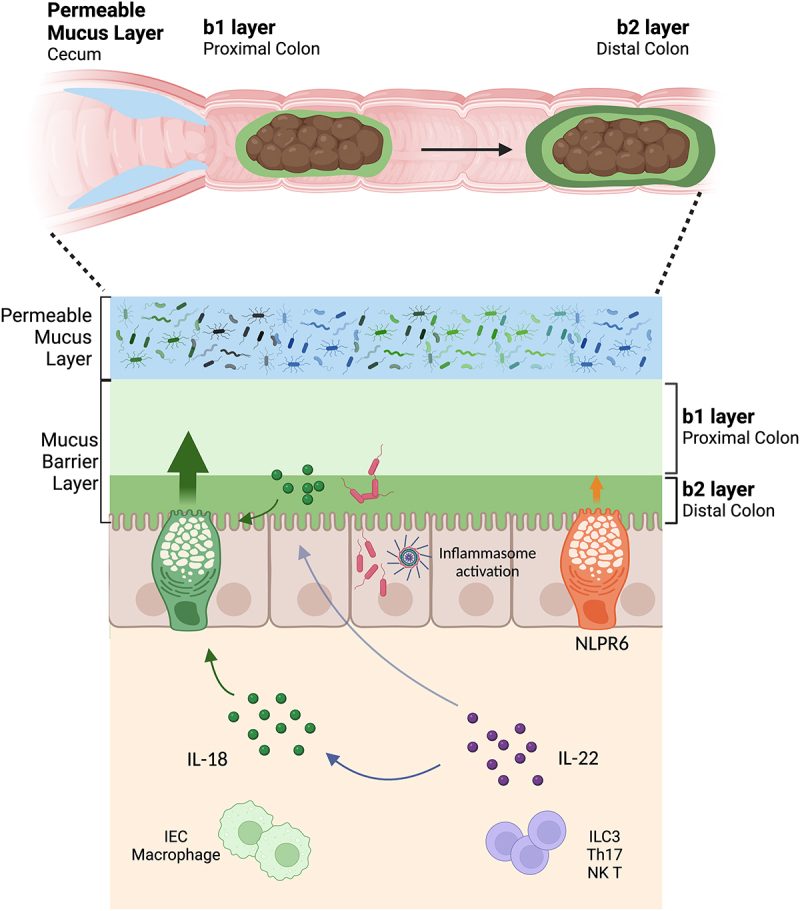
Inflammasome-dependent responses in the gut limit *S*. Typhimurium burdens. Schematic summarizing the goblet cell-associated responses described in this manuscript. Note that IL-18 may be produced by IECs and/or macrophages in response to *S*. Typhimurium infection, while IL-22 may be produced by ILC3, Th17 or NK T cells.

The intestinal mucosa is the primary target for *S*. Typhimurium, as well as other enteric pathogens, when they launch their assaults on mammalian hosts. Our research shows that inflammasome activation is a key initiator of the host’s intestinal mucosal response during the early stages of *S*. Typhimurium infection, promoting the secretion of IL-22 and IL-18. These cytokines drive mucin secretion from goblet cells in the cecum and proximal colon, likely aimed at protecting the mucosal surface from further infection by invading pathogens (see Figure 9). Correspondingly, the absence of IL-18 or IL-22 significantly impaired mucin release, whereas the Nlrp6 inflammasome did not impact mucin release in this region, but instead, modestly contributed to mucin secretion in the distal colon through IL-22 and IL-18 independent pathways.

Previous research has shown that inflammasome signaling is involved in the early defense of the intestinal mucosa against *S*. Typhimurium, through the expulsion of infected IECs.^3,6–8^ *Casp1/11*^−/−^ mice are defective for inflammasome-dependent IEC expulsion, leading to increased intra-epithelial proliferation of *S*. Typhimurium. While these intracellular *S*. Typhimurium clearly contribute to the heavier pathogen burdens found in *Casp1/11*^−/−^ mice,^3,7^ our observations suggested these heavily infected IECs cannot on their own explain the dramatically higher pathogen burdens carried in the gut of these mice, as compared to their WT controls. Thus, inflammasome signaling must drive other protective mechanisms that limit pathogen burdens in the intestinal lumen and mucosal surface.

While studies in *Casp1/11*^−/−^ mice demonstrated that inflammasome signaling is not required to maintain the colonic mucus layer under homeostatic conditions,^38^ our results showed that upon *S*. Typhimurium infection, inflammasome signaling drives a massive release of Muc2 into the cecal lumen. This is accompanied by increased mucin release in the proximal and distal regions of the colon, where it contributes to an increase in the thickness of the mucus barrier layer, as distinguished using MALII staining. Previous studies determined that pyroptosis and expulsion of IECs during *S*. Typhimurium infection was due to IEC-intrinsic inflammasome signaling.^7,8^ In contrast, the widespread release of mucin from goblet cells in both proximal and distal regions of the colon suggests a more systematic response involving multiple cell types and soluble mediators in the gut.

Colonic tissue explants were previously used to identify a novel goblet cell subset, termed “sentinel goblet cells”, that respond to the presence of nearby bacterial products by rapidly secreting mucins, as well as inducing mucin release by nearby canonical goblet cells, through the activation of caspase-1 and −11 via the Nlrp6 inflammasome.^19,27^ Considering the exaggerated susceptibility of *Nlrp6*^−/−^ mice to enteric *S*. Typhimurium infection, we initially suspected this pathway would be a major contributor to the mucin release seen in our infection model. Instead of promoting mucin release in the cecum and proximal colon, Nlrp6 signaling only modestly increased mucin release from goblet cells in the distal colon, thereby thickening the b2 mucus layer. Moreover, the Nlrp6-dependent mucin secretion seen in the *S*. Typhimurium infection model appeared to be independent of IL-18 levels, despite the ability of the Nlrp6 inflammasome to process IL-18.^39^ These findings suggest Nlrp6 likely controls pathogens burdens by other, yet to be defined, mechanisms. The role of Nlrp6 in host defense against bacterial infections remains poorly understood, as *Nlrp6*^−/−^ mice were previously shown to be protected against intra-peritoneal *S*. Typhimurium infection, with Nlrp6 acting as a negative regulator of innate immunity.^40^ Another recently identified goblet cell subtype within the distal colon are the UEA1^+^ WGA^+^ inter-crypt goblet cells that also secrete mucins in response to external stimuli.^41^ These goblet cells could potentially contribute to the increased mucin secretion seen in the distal colon during *S*. Typhimurium infection, however, we did not observe fewer mucin-depleted UEA1^+^ goblet cells in infected *Nlrp6*^−/−^ mice. Thus, further investigation is needed, both to clarify whether sentinel and inter-crypt goblet cells contribute to the mucin release observed in the distal colon during *S*. Typhimurium infection, as well as to characterize the goblet cells in the cecum/proximal colon that respond to IL-18 during infection.

Loss of IL-18 led to increased *S*. Typhimurium burdens, similar to those observed in *Casp1/11*^−/−^ mice. IL-18 has been previously shown to reduce intestinal pathogen burdens by promoting *Ifnγ* production and by stimulating the shedding of infected pyroptotic cells.^33,42,43^ Our study focused on its impact on intestinal goblet cells, as previous studies have shown IL-18 can both promote and inhibit goblet cell maturation and mucin secretion, depending on the model tested.^22,36,44^ For example, elevating levels of circulating IL-18 over the course of DSS-induced colitis resulted in worsened colitis and significant loss of goblet cells.^22^ Moreover, isolation stress-induced increases in IL-18 production also promote goblet cell depletion in the recta of mice.^44^ In contrast, mouse ileal enteroids supplemented with IL-18 showed increased budding and higher *Muc2* expression, while adherent invasive *E. coli* (AIEC) infection of *Il18*^−/−^ mice impairs mucin secretion in the small intestine.^36^ We found IL-18 to be vital in promoting mucin secretion in the proximal colon, as we noted a reduced thickening of the b1 mucus layer in the distal colons of infected *Il18*^−/−^ mice, whereas the b2 mucus layer remained consistent between *Il18*^−/−^ mice and their littermates. Supplementation of recombinant IL-18 in *Il22*^−/−^ mice drastically increased mucin production and secretion, confirming IL-18’s role in promoting goblet cell maturation and mucin secretion. These data support our finding that the injection of exogenous IL-18 into uninfected mice did not increase baseline mucus levels and suggests IL-18 may require other infection associated factors to cause the observed increases in mucin production and secretion. We note that although baseline secretion levels of IL-18 were similar in *Casp1/11*^*+/+*^, *Nlrp6*^*+/+*^ and *Il18*^*+/+*^ mice, the levels detected in uninfected C57BL/6 mice were substantially greater, despite displaying asimilar baseline colonic mucus layer thickness as the other WT mice.

The cytokine IL-22 has also been linked to goblet cell development and function in the intestinal mucosa.^21,33–35^ *Il22*^−/−^ mice display reduced goblet cell numbers after DSS-induced colitis or during infection with the intestinal helminth *Trichuris muris*.^21,28^ Exogenous IL-22 was shown to ameliorate DSS colitis by aiding in goblet cell reconstitution and mucin production,^34^ while in another study, it protected mice during *S*. Typhimurium infection by upregulating *Fut2* and the α −1,2-fucosylation of proteins and lipids in the intestinal mucosa.^45^ The antimicrobial lectins Reg3γ and Reg3β are also upregulated in response to IL-22 signaling.^46^ Notably, we found that *Il22*^−/−^ mice showed limited mucin release in response to *S*. Typhimurium infection, particularly from the cecum and proximal colon, while *Fut2* and *Reg3γ* expression positively correlated with IL-22 levels, highlighting IL-22’s role in promoting host defense against bacterial infections targeting the intestinal mucosa.

Several studies have shown that IL-22 is closely linked to inflammasome activation and the subsequent activation/release of IL-18.^47,48^ For example, IL-22 treatment of intestinal organoids increases the expression of the inflammatory caspases (*Casp1*, *Casp4* and *Casp5*) while IL-18 can also potentiate IL-22 signaling to trigger protective IEC proliferation during wound healing.^46,47^ In turn, the production of IL-22 is also crucial for maintaining elevated IL-18 levels during infections by the parasite *Toxoplasma gondii* or the bacterial pathogen *Citrobacter rodentium*, creating a positive feedback loop.^49^ However, other studies have suggested a unidirectional signaling pathway between IL-22 and IL-18, where IL-22 supplemented to *Il18*^−/−^ mice during AIEC infection failed to increase goblet cell numbers or mucin secretion in the colon, whereas IL-22 supplementation in WT mice did exert these effects.^36^ Additionally, an inverse relationship between inflammasome signaling and IL-22 expression has been demonstrated, where *S*. Typhimurium-infected group 3 innate lymphoid cells (ILC3) cells undergo pyroptosis after inflammasome activation, leading to reduced IL-22 production at later stages of infection.^48^ In our acute *S*. Typhimurium infection model, we found that IL-18 levels are modulated by IL-22, but not the reverse, where the supplementation of IL-18 to *Il22*^−/−^ mice during infection was able to restore mucin secretion. Hence, during the early phase of infection, IL-22 can unidirectionally upregulate IL-18 levels at the primary site of infection, allowing IL-18 to promote goblet cell maturation and subsequent mucin secretion, potentially to help flush *S*. Typhimurium down the GI tract.

Overall, these findings demonstrate the crucial role played by inflammasome signaling in promoting enteric mucosal defense during acute *S*. Typhimurium infections, while also shedding light on the multifaceted functions of the IL-22/IL-18 signaling pathway and the key role these cytokines play in promoting mucin secretion. While our earlier studies on IEC shedding focused on IEC intrinsic inflammasomes,^3,7^ the key roles of caspase-1 and −11, and IL-22 and IL-18, in mucin release highlights the involvement of multiple cell types. While IECs are known to produce IL-18, so do many other innate immune cells in the gut, such as macrophages,^50^ while IL-22 can be produced by ILC3 as well as T lymphocytes^51^ (see Figure 9). Our findings should help to clarify the often-contradictory description of the roles played by these cytokines in modulating IEC function during intestinal inflammation. Moreover, these findings highlight that although several unique goblet cell subsets (sentinel and inter-crypt) have been identified in the distal colon, the host may still rely on canonical goblet cells in the cecum and proximal colon to respond to enteric pathogen infections with increased mucin release.

## Materials and methods

### Mouse strains and S. Typhimurium infections

*Casp1/11*^−/−^ mice (*Ice*^−/−^ or *Casp1*^−/−^*Casp11*^null/null^) were obtained from Genentech, *Nlrp6*^−/−^ mice were a kind gift from Dr. Gabriel Núñez, University of Michigan. *Il18*^−/−^ mice^52^ were purchased from Jackson Laboratories mice. *Il22*^−/−^ mice were provided by Genentech. Except for the *Il22*^−/−^ mice, all mice were bred from heterozygous breeders resulting from backcrossed KO with C57BL/6NCrl mice purchased from Charles River. All resulting litters were genotyped using the primers listed in Table 1:

**Table 1.** Genotyping primers.

*Casp1/11*^−/−^	FWD - GGT CTT GTC TCT TAT AGG AGA TGG
	REV - GGA ATC AAC CCCAAA CAC TGA AGA
*Nlrp6*^−/−^	FWD - CTC AGA CGC TGT GGA CCT TG
	REV - CTC ACA CGG AGA GAA GCA CC
*Il18*^−/−^	FWD (WT) - GGC AGC AAG CAC TCT TAA CC
	FWD (Mutant) - AAT TCG CCA ATG ACA AGA CG
	REV (Common) – ACA AAC CCT CCC CAC CTA AC

All mice (8–12 weeks old, female) were bred under specific pathogen-free conditions at the BC Children’s Hospital Research Institute (BCCHRI). For oral infections, mice were gavaged with streptomycin (100 mg/kg) 24 h before infection, then gavaged with an overnight LB culture of wild-type *S*. Typhimurium SL1344 (naturally streptomycin-resistant,^53^) diluted 1/100 in PBS (~2.5 × 10^6^ CFU). Mice were euthanized at 24 h p.i. For the IL-18 supplementation experiments, mice were injected intraperitoneally (i.p.) at 6 h p.i. with 2 μg of mouse recombinant IL-18 (Biolegend). For the IL-22 neutralization experiments, mice were injected i.p. 1 h before infection with 100 µL dose of IL-22 (150 µg αIL-22; 8e11, Genentech) or the equivalent dose of mouse isotype-matched control antibody (ragweed; 10D9.1E11.1F12; Genentech). Two to four independent experiments were performed for all genotypes mentioned. All mouse experiments were performed according to protocols approved by the University of British Columbia’s Animal Care Committee and in direct accordance with the Canadian Council on Animal Care (CCAC) guidelines.

### Pathogen burdens

Mice were anesthetized with isoflurane and euthanized via cervical dislocation. For pathogen enumeration, cecal and colonic tissues were collected and homogenized separately in 1 ml of sterile PBS. Samples were serially diluted and plated on streptomycin-supplemented (100 µg/ml) LB agar plates and incubated at 37°C overnight. Colonies were then enumerated and normalized to the weight of each tissue.

### Histology and goblet cell scoring

Tissue samples for histology and immunohistochemistry staining were fixed either in 10% neutral buffered formalin (Fisher Scientific) overnight and transferred to 70% ethanol or fixed in Methacarn (60% dry methanol, 30% chloroform, 10% glacial acetic acid) overnight, washed with methanol and transferred to 100% ethanol. All fixed tissue was embedded in paraffin and sectioned into 5 μm thick sections.

Formalin-fixed tissue sections were stained with hematoxylin and eosin (H&E) (performed by the BCCHRI Histology Core Lab) and imaged and scored by blinded observers. Tissue sections were assessed for overall pathological damage and goblet cell depletion through quantification of goblet cell number and size (i.e. diameter). Three images were taken for each tissue section at × 200 magnification using Zeiss Axio Imager with Zen Pro software. For goblet cell numbers, five crypts per image were selected at random to count goblet cell numbers in each crypt. All analysis was performed using ImageJ.

### Immunofluorescent staining and mucus thickness analysis

Paraffin-embedded tissues were deparaffinized by heating to 60°C for 15 min, cleared with xylene, and rehydrated through an ethanol gradient to water. Antigen retrieval was performed in steam-heated citrate buffer for 30 mins, before cooling to room temperature and washing with water. Tissues were treated in PBS, 0.1% Triton (v/v) X-100 and 0.05% (v/v) Tween 20 for 15 mins, then blocked with 5% (v/v) donkey serum in PBS, 0.01% (v/v) Triton X-100 and 0.05% (v/v) Tween 20. Primary antibodies used were anti-Muc2 (1:200; Bolster), anti-RegIIIβ (1:50; Biotechne R&D), and anti-E-cadherin (1:200, BD Biosciences). Lectins were UEA-1 (1:500; Vector Laboratories) and MALII (1:500; Vector Laboratories). Tissues were then probed with Alexa Fluor 488-conjugated donkey anti-goat IgG (1:2000; Life Technologies), Alexa Fluor 568-conjugated donkey anti-rabbit IgG (1:2000; Life Technologies), and/or Alexa Fluor 680-conjugated donkey anti-sheep IgG (1:2000; Life Technologies). Tissues were mounted using ProLong Gold Antifade reagent (Life Technologies) containing DAPI for DNA staining.

Stained sections were viewed on a Zeiss AxioImager microscope. To quantify mucus layer thickness, three random images were taken per section for Muc2- and MALII-immunostained, Methacarn-fixed colonic sections, and two measurements were taken per image for each Muc2^+^ section (total mucus layer thickness), MALII^+^ section (b2 mucus layer), MALII^−^ section (b1 mucus layer).

### RNA isolation and RT-PCR

Immediately following euthanization of mice, cecal tissues were collected, washed 3× in PBS and placed in RNAlater (Qiagen), incubated at 4°C overnight, then stored at − 80°C. Total RNA was extracted and DNase-treated using a RNeasy Mini Kit (Qiagen) according to the manufacturer’s instructions. Total RNA was quantified using a NanoDrop microvolume spectrophotometer, and cDNA was synthesized using 0.5 μg of total RNA with 5× All-In-One RT MasterMix (Applied Biological Materials (abm)). For the RT-PCR reaction, 5 μl of a 1:5 dilution of cDNA was added to 10 μl Bio-Rad SYBR Green Supermix with primers (final concentration, 300 nM; final volume, 20 μl), and RT-PCR was carried out using a Bio-Rad MJ MiniOpticon machine. Primer sequences are listed in Table 2:

**Table 2.** RT-PCR primers for gene expression analysis.

*Reg3γ*	FWD – TCC CAG GCT TAT GGC TCC TA
	REV – GCA GGC CAG TTC TGC ATC A
*Fut2*	FWD – ACC TCC AGC AAC GAA TAG TGA
	REV – GCC GAT GGA ATT GAT CGT GAA
*Ifnγ*	FWD – ATG AAC GCT ACA CAC TGC ATC
	REV- CCA TCC TTT TGC CAG TTC CTC
*Rplp0*	FWD – AGA TTC GGG ATA TGC TGT TGG C
	REV – TCG GGT CCT AGA CCA GTG TTC

CFX Maestro software version 1.1 (Bio-Rad) was used for data quantification. All gene expression data were normalized to the housekeeping gene *Rplp0*, and fold change for each gene was calculated by setting one sample from PBS-treated WT group as “1” and normalizing other samples accordingly.

### Ex vivo secretion and ELISA assays

Cecal tissue (0.5–1 cm) was excised from each mouse, washed extensively in PBS then stored on ice briefly in *ex vivo* secretion medium (RPMI (Gibco), 10% FBS (Sigma-Aldrich), 100 U/ml penicillin-streptomycin, (Gibco), 1 mM sodium pyruvate (Gibco), 1×MEM non-essential amino acids (Sigma-Aldrich), 100 µg/ml gentamicin (Gibco)). Ceca and secretion medium were transferred under sterile conditions to a 24-well plate for 24 h incubation at 37°C with 5% CO_2_. Media was then collected and centrifuged at 10,000 rpm and 4°C, supernatant collected and stored at −80°C.

Before ELISA assays, the protein concentration in each sample was quantified using the Pierce 660 assay (ThermoFisher) according to the manufacturer’s instructions. All samples were diluted to 0.5 µg/µl of total protein, before undergoing IL-22 ELISA (Murine IL-22 ELISA MAX™ Deluxe Set; BioLegend) or IL-18 ELISA (Mouse IL-18 DuoSet ELISA; Biotechne R&D) in duplicates following the manufacturer’s instructions.

### Muc2 dot blot assay

Luminal contents from the ceca or distal colons of mice were collected in PBS containing protease inhibitor (Roche) and stored at −80°C until processing. After thawing, samples were homogenized by sonification and quantified for protein concentration using the Pierce 660 assay (ThermoFisher). For each sample, 5 ng of total protein was spotted onto nitrocellulose membrane and left to air dry for 20 minutes. The membranes were then blocked in 5% (w/v) bovine serum albumin (BSA; Sigma) in Tris Buffered Saline with 0.1% (v/v) Tween 20 (TBST) for 1 h, incubated with rabbit anti-Muc2 antibodies (1:500; Bolster) in 5% BSA in TBST for 1 h, washed twice in TBST for 10 min each, incubated with anti-rabbit HRP (1:2000; Bolster) in 5% BSA in TBST for 1 h, then washed again twice in TBST for 10 min each. The blots were incubated with Enhanced Chemiluminescence (ECL; ThermoFisher) for 5 min and imaged using a BioRad ChemiDoc.

## Statistical analysis

All results presented in this study are expressed as mean values ± standard deviations (SD). Student’s t-tests or one-way analysis of variance (ANOVA) tests followed by Tukey’s multiple comparison post-hoc test were used to determine statistical significance using GraphPad Prism software, version 10.1.0 for Mac. A p-value of 0.05 or less was considered significant, with asterisks denoting statistical significance in Figures.

## Supplementary Material

Supplemental Material

## Data Availability

Supporting data are available from the corresponding authors, LAK and BAV, upon reasonable request.
