# Supplementary material for: Inflammasome activation links enteric Salmonella Typhimurium infection to a rapid, cytokine-dependent increase in intestinal mucin release
Source: Gut Microbes. 2024 Oct 20;16(1):2413372. doi: 10.1080/19490976.2024.2413372 (PMC11497969; doi:10.1080/19490976.2024.2413372)
Supplement: Supplemental Material [file KGMI_A_2413372_SM2763.zip › Final Supp Figures Sept6_LAK.docx]

**
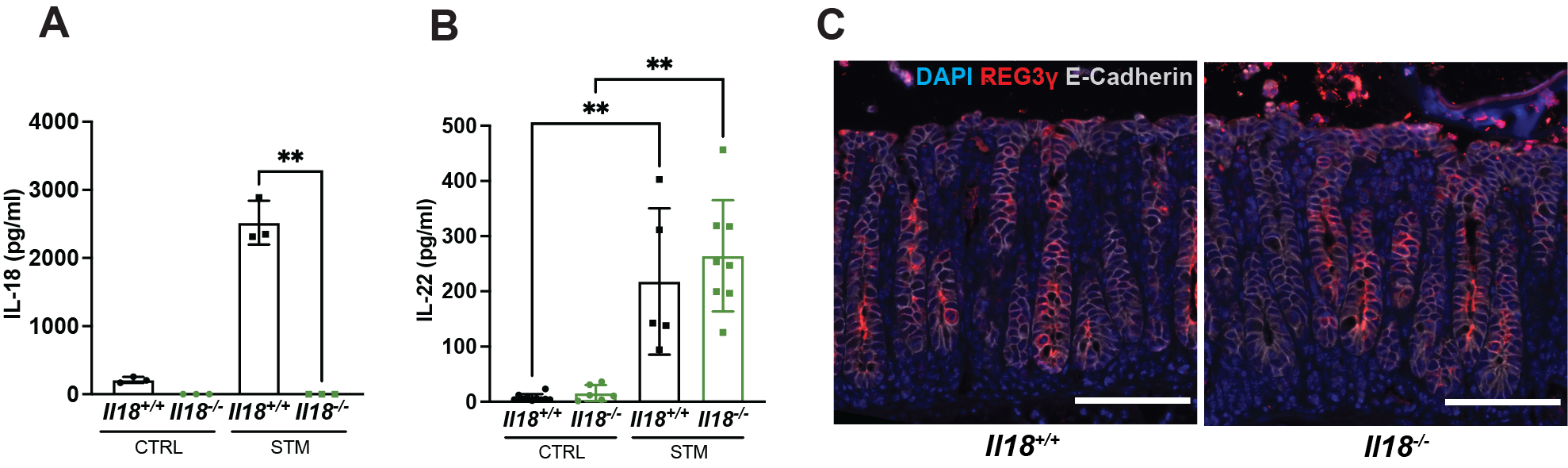
Supplementary Figure 1. Inflammatory responses in IL-18 deficient mice.** *Ex vivo* secretion of (A) IL-18 and (B) IL-22 from ceca of baseline control (CTRL) and infected (STM) *Il18^+/+^* (black) and *Il18^-/-^* littermates (green) were quantified by ELISA. Data shown as mean ± SD. Stastical significance was determined by one-way ANOVA. ** = p < 0.01. (C) Immunofluorescence staining of cecum from *S*. Typhimurium-infected *Il18^+/+^* and *Il18^-/-^* littermates. Nuclei (blue), Reg3γ (red), E-Cadherin (white). Scale bars represent 100 μm.

***
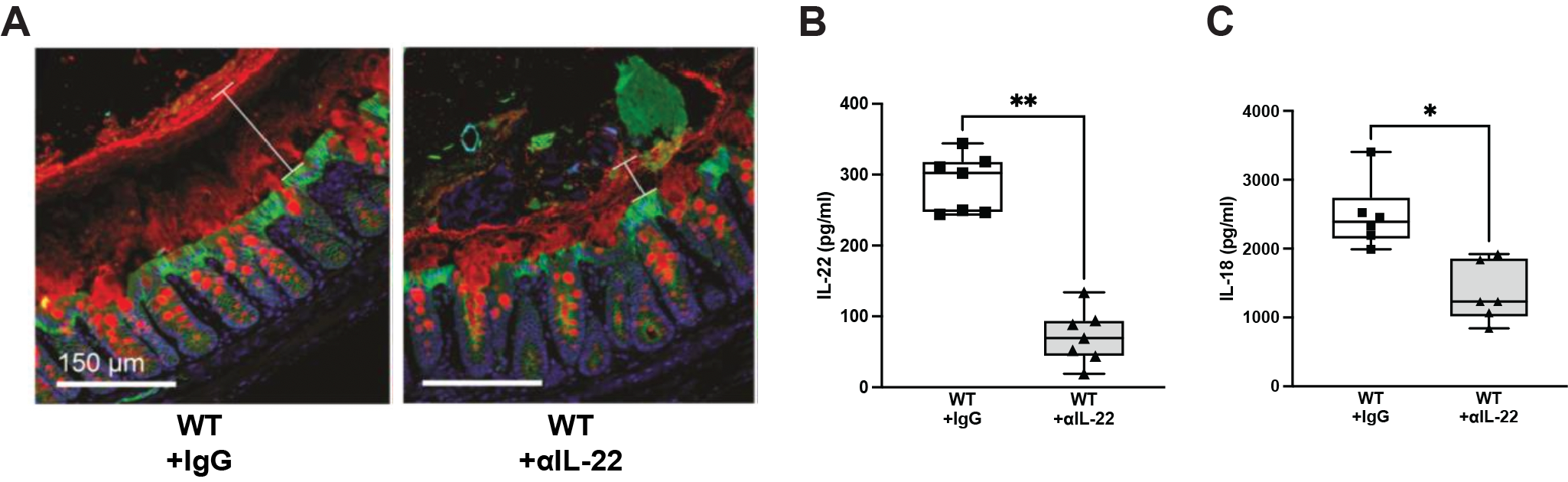
*Supplementary Figure 2. Inhibition of IL-22 leads to reduced colonic mucus thickness.** (A) Immunofluorescence staining of the distal colon of infected mice pre-treated with IgG control or αIL-22 via intraperitoneal injection 1 h prior to *S*. Typhimurium infection (n≥5 for each group pooled from two independent experiments). Nuclei (blue), Muc2 (red), E-cadherin (green). Note the diminished mucus layer in infected mice that received αIL-22. Scale bars represent 150 μm. *Ex vivo* secretion of (B) IL-22 and (C) IL-18 from ceca of infected mice was quantified by ELISA. Data shown as mean ± SD. Statistical significance was determined by one-way ANOVA. *p < 0.05; **p < 0.01.

**
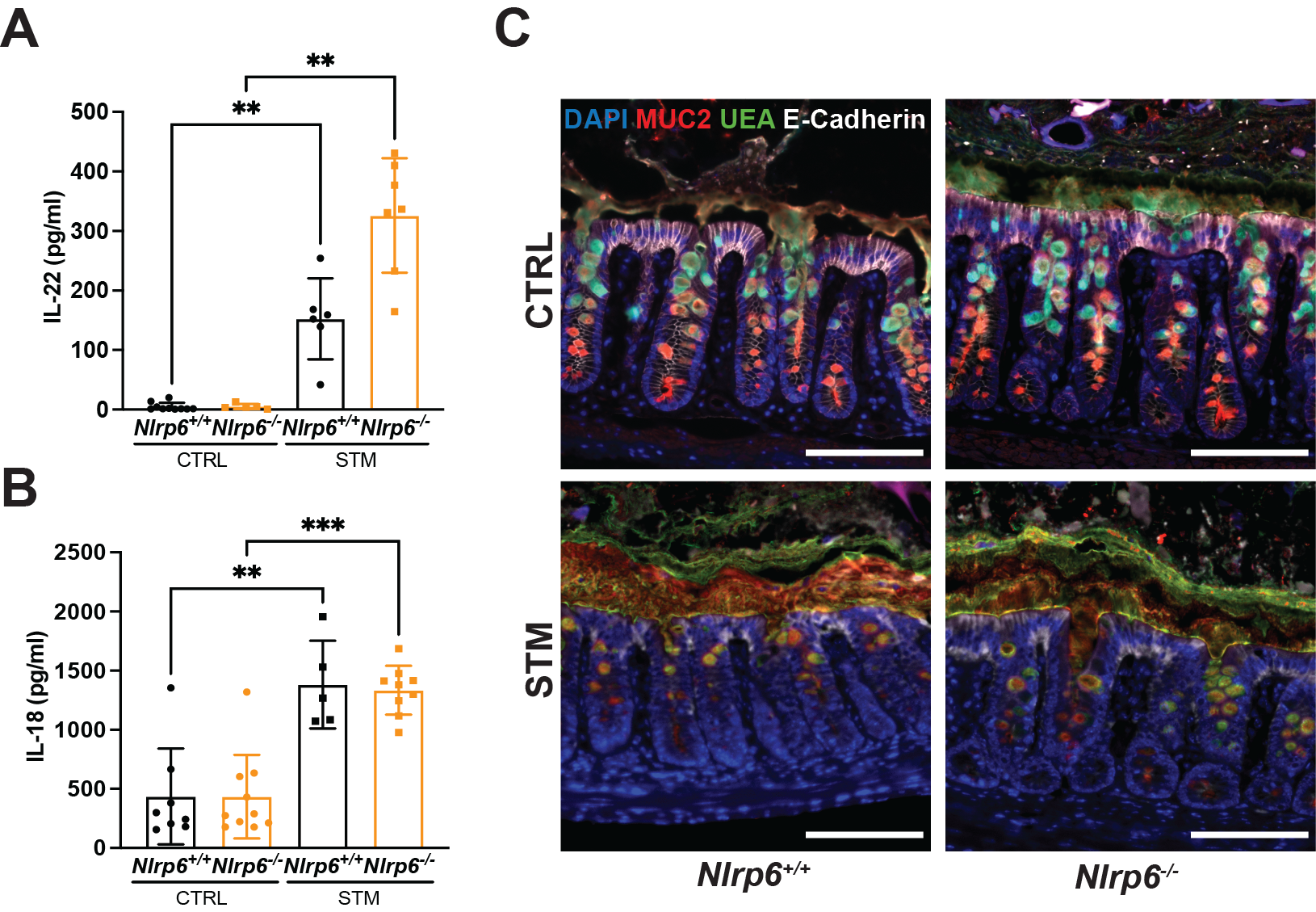
Supplementary Figure 3. Nlrp6-dependent inflammatory responses during *S.* Typhimurium infection.** *Nlrp6^+/+^* and *Nlrp6^-/-^* littermate mice were infected with *S.* Typhimurium for 24 h. *Ex vivo* secretion of (A) IL-22 and (B) IL-18 from the ceca of baseline control (CTRL) and infected (STM) mice was quantified by ELISA. Data shown as mean ± SD. Statistical significance was determined by one-way ANOVA. **p < 0.01; ***p < 0.001. (C) Immunofluorescence staining of baseline control and infected distal colons. Nuclei (blue), Muc2 (red), UEA (green), E-cadherin (white). Scale bars represent 100 μm.
